# Coenzyme Q10 Ameliorates Chemotherapy-Induced Neurotoxicity in iPSC-Derived Neurons by Reducing Oxidative Stress

**DOI:** 10.3390/ijms26199647

**Published:** 2025-10-02

**Authors:** Nidaa A. Ababneh, Razan AlDiqs, Mohammad H. Gharandouq, Mohammad A. Ismail, Raghda Barham, Fairouz Nairat, Omar Hamdan, Qais Mussa, Momen Sarhan, Amira T. Masri, Anas Abu-Humaidan, Sofian Al Shboul, Areej Abuhammad, Abdalla Awidi, Tareq Saleh

**Affiliations:** 1 Cell Therapy Center, The University of Jordan, Amman 11942, Jordan; mohd.gharandouq@outlook.com (M.H.G.); mohd.ismail2@yahoo.com (M.A.I.); raghda.barham@gmail.com (R.B.); fairouzomarnairat@gmail.com (F.N.); momensarhan0@gmail.com (M.S.); abdalla.awidi@gmail.com (A.A.); 2 Department of Allied Sciences, Faculty of Arts and Sciences, Al-Ahliyya Amman University, Amman 19328, Jordan; razan.nezar.97@gmail.com; 3 South Australian ImmunoGENomics Cancer Institute, Adelaide Medical School, University of Adelaide, Adelaide, SA 5005, Australia; 4 School of Medicine, The University of Jordan, Amman 11942, Jordan; amr0193232@ju.edu.jo (O.H.); qaismussa@gmail.com (Q.M.); 5 Department of Paediatrics, Division of Child Neurology, School of Medicine, The University of Jordan, Amman 11942, Jordan; masriamira69@hotmail.com; 6 Department of Pathology, Microbiology and Forensic Medicine, School of Medicine, The University of Jordan, Amman 11942, Jordan; a.abuhumaidan@ju.edu.jo; 7 Department of Pharmacology and Public Health, Faculty of Medicine, The Hashemite University, Zarqa 13133, Jordan; sofian@hu.edu.jo; 8 Department of Pharmaceutical Sciences, School of Pharmacy, The University of Jordan, Queen Rania Street, Amman 11942, Jordan; a.abuhammad@gmail.com; 9 Hemostasis and Thrombosis Laboratory, School of Medicine, The University of Jordan, Amman 11942, Jordan; 10 Department of Hematology and Oncology, Jordan University Hospital, Amman 11942, Jordan; 11 Department of Pharmacology & Therapeutics, College of Medicine & Health Sciences, Arabian Gulf University, Manama 329, Bahrain

**Keywords:** Coenzyme Q10 (CoQ10), chemotherapy-induced neurotoxicity (CIN), reactive oxygen species (ROS), human induced pluripotent stem cells (hiPSCs), neuroprotection

## Abstract

Chemotherapy-induced neurotoxicity (CIN) is a major barrier against optimal anticancer treatment. This study investigated the neuroprotective effects of the naturally occurring antioxidant, Coenzyme Q10 (CoQ10), against CIN using a model of induced pluripotent stem cell (iPSC)-derived neurons. iPSCs have consistently proven to be reliable for disease modeling and drug discovery. We employed cell viability, oxidative stress, and mitochondrial function assays to measure the effect of 10 μM CoQ10 on iPSC-derived motor neuron progenitors (iPSC-MNPs) that were exposed to five chemotherapeutic agents: 5-Fluorouracil, methotrexate, paclitaxel (0, 1, and 10 μM) and doxorubicin, and vincristine (0, 0.1, and 1 μM). Our findings show that CoQ10 significantly reversed the reduction in cell viability inflicted by the exposure of iPSCs-MNPs to all five chemotherapeutics. Moreover, CoQ10 treatment resulted in a marked reduction in intracellular ROS levels and enhancement of mitochondrial membrane potential (MMP) in a drug- and dose-dependent manners, highlighting its role in preserving mitochondrial health. This study is the first to explore the protective effects of CoQ10 against CIN using an iPSC-derived neuronal platform, offering insights into its potential therapeutic use. Further investigation is essential to validate these findings and to determine the behavioral effects of CoQ10 in in vivo models of CIN.

## 1. Introduction

Chemotherapy remains a cornerstone of cancer treatment; its success is often limited by damage to noncancerous cells [1]. Chemotherapy-induced neurotoxicity (CIN) is a common adverse effect associated with several chemotherapeutic agents. It is clinically linked to debilitating conditions such as chemotherapy-induced peripheral neuropathy (CIPN) and chemotherapy-induced cognitive impairment (CICI), both of which significantly affect patients’ quality of life. Studies show that approximately 30–40% of patients treated with neurotoxic chemotherapy develop CIPN [2], while up to 75% of patients develop CICI during treatment, with 35% continuing to experience CICI symptoms several months after chemotherapy [3]. CIPN typically presents with sensory disturbances (including pain) and motor dysfunction, primarily affecting the hands and feet [4,5]. The pathophysiology of CIPN is complex and multifactorial, involving mitochondrial dysfunction, oxidative stress, neuroinflammation, and impaired axonal transport [6,7]. Although CIPN is common, effective preventive strategies remain a major unmet clinical need in oncology, indicating the need to investigate CIN mechanisms contributing to CIPN.

In recent years, the development of induced pluripotent stem cells (iPSCs) has revolutionized biomedical research, offering a new platform to model human diseases in vitro. iPSCs, derived from reprogrammed somatic cells, possess the ability to differentiate into various cell types, including peripheral neurons, making them highly valuable for studying CIPN [8]. By using iPSC-derived peripheral neuron models, the effects of chemotherapeutic agents on human neurons can be investigated in a controlled in vitro environment, thus providing insights into underlying mechanisms of CIPN, such as mitochondrial dysfunction, oxidative damage, and inflammation [4,5,7]. Additionally, the ability to generate disease-specific or patient-specific iPSC lines significantly enhances the translational potential of these approaches and facilitates the identification of more effective pharmacological interventions.

Natural compounds are increasingly explored as supportive strategies in cancer therapy due to their multitargeted effects and reduced toxicity [9,10]. Coenzyme Q10 (CoQ10), a naturally occurring antioxidant and a vital component of the mitochondrial electron transport chain, has gained increasing attention as a promising agent for mitigating neurotoxicity [11,12,13,14,15]. CoQ10 plays a critical role in maintaining mitochondrial function and protecting cells from oxidative damage and lipid peroxidation [16]. Its neuroprotective effects have been demonstrated in various models of neurological disorders, including Parkinson’s disease and Alzheimer’s disease [15,17,18]). These findings suggest that CoQ10 may also offer therapeutic potential for CIPN, although its efficacy in this context has not been fully investigated.

In this study, we utilized iPSC-derived motor neurons (iPSC-MNPs) to investigate the neuroprotective potential of CoQ10 against CIN. We evaluated CoQ10’s protective effect against five chemotherapeutic agents, namely, 5-fluorouracil, methotrexate, paclitaxel, doxorubicin, and vincristine, all of which have been associated with CIN to varying extents. We also assessed CoQ10’s impact on oxidative stress and mitochondrial function due to their known involvement in CIPN pathogenesis. Our findings demonstrate that CoQ10 offers significant protection against chemotherapeutic neurotoxicity in vitro, suggesting its potential as a therapeutic strategy for the mitigation of CIN-related compilations.

## 2. Results

### 2.1. Confirmation of Pluripotency in iPSC Lines

**Figure 1:**
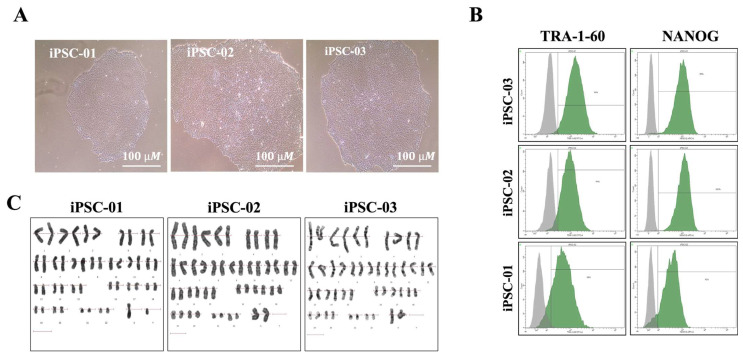
Morphology, pluripotency marker expression, and karyotype analysis of the iPSC lines. (**A**) Representative phase-contrast images of three independent iPSC lines (iPSC-01, iPSC-02, and iPSC-03), showing typical human pluripotent stem cell colony morphology. (**B**) Flow cytometry analysis of pluripotency-associated markers TRA-1-60 and NANOG (green histograms) compared with isotype controls (gray), confirming successful reprogramming and maintenance of pluripotency in all lines. (**C**) Karyotype analysis demonstrated a normal chromosomal complement (46, XY or 46, XX) in all three clones, indicating genomic stability during reprogramming.

This study aimed to investigate the neuroprotective effect of CoQ10 against CIN in iPSC-derived motor neuron progenitors (iPSC-MNPs). To ensure the reliability of our model, we first characterized three iPSC lines previously derived from HDFs [19,20,21]. Pluripotency and differentiation potential were confirmed through genetic stability assessments and flow cytometry, which demonstrated strong expression of pluripotency markers (TRA-1-60 and NANOG) [22]. Karyotyping confirmed chromosomal stability across all lines. We then evaluated their differentiation into motor neurons, followed by assessing the effects of CoQ10 on cell viability, ROS generation, and mitochondrial membrane potential (MMP) after treatment with chemotherapeutic agents. The pluripotency of our iPSC lines (JUCTCi011-A (iPSC-01), JUCTCi010-A (iPSC-02) and JUCTCi010-B (iPSC-03) was confirmed by morphological features (Figure 1A) and immunophenotypic staining of key pluripotency markers, including TRA-1-60 and NANOG, both of which showed high expression levels (>90%) (Figure 1B). Karyotype analysis confirmed chromosomal stability, with all three lines exhibiting a normal karyotype with no detectable abnormalities (Figure 1C).

### 2.2. Confirmation of iPSC Differentiation into Motor Neurons (MNs)

**Figure 2:**
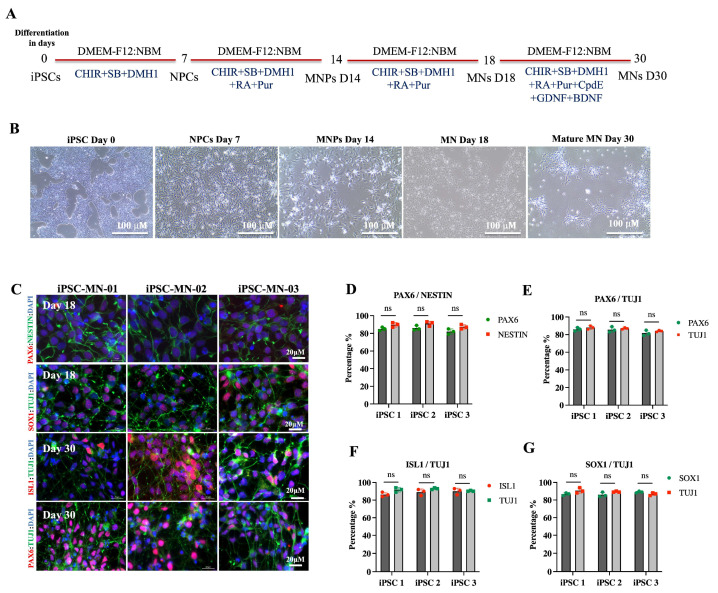
Differentiation of iPSCs into MNs. (**A**) Schematic timeline of iPSC differentiation into MNs (**B**) Representative morphology at: day 0 iPSCs exhibit tightly packed colonies with well-defined borders. By day 18, differentiating cells extend neurite-like projections, and by day 30, mature neurons form neuronal networks with visible connections. (**C**) Immunostaining of MNPs at day 18 and mature MNs at day 30, confirming differentiation with markers NESTIN, TUJ1, SOX1, ISL1, and PAX6, with DAPI counterstaining. (**D**–**G**) Quantification of marker expression, showing comparable levels across the three iPSC lines. ns = not significant.

The differentiation of iPSCs into MNs was initiated using a basal medium composed of DMEM-F12 and neurobasal medium (NBM), supplemented with various stage-specific growth factors at specific time points (days 0, 7, 14, and 30) as described previously [21]. iPSCs began differentiation into MNPs by day 18 with further maturation, and the establishment of neuronal networks observed by day 30 (Figure 2A,B). By day 18, neurite projections were evident, and by day 30, neuronal connections were established (Figure 2B). Further confirmation of iPSC differentiation into MNs was achieved using fluorescent confocal microscopy. Immunostaining was performed on day 18 MNPs and day 30 mature MNs with antibodies against neuronal markers including NESTIN, TUJ1, SOX1, ISL1, and PAX6 (Figure 2C). Quantitative analysis of marker expression showed consistent levels at both day 18 and day 30 MNs, with no significant differences detected across the examined markers (Figure 2D–G). Validation of motor neuronal identity at day 18 was further supported by staining for two general neuronal markers (PAX6 and Nestin) and two mature motor neuron markers (ISL1 and SMI32), all of which demonstrated robust positive signals across treatments (Supplementary Figure S1).

### 2.3. Potential Protective Effects of CoQ10 on iPSC-MNPs Viability Following Exposure to Chemotherapeutic Agents

**Figure 3:**
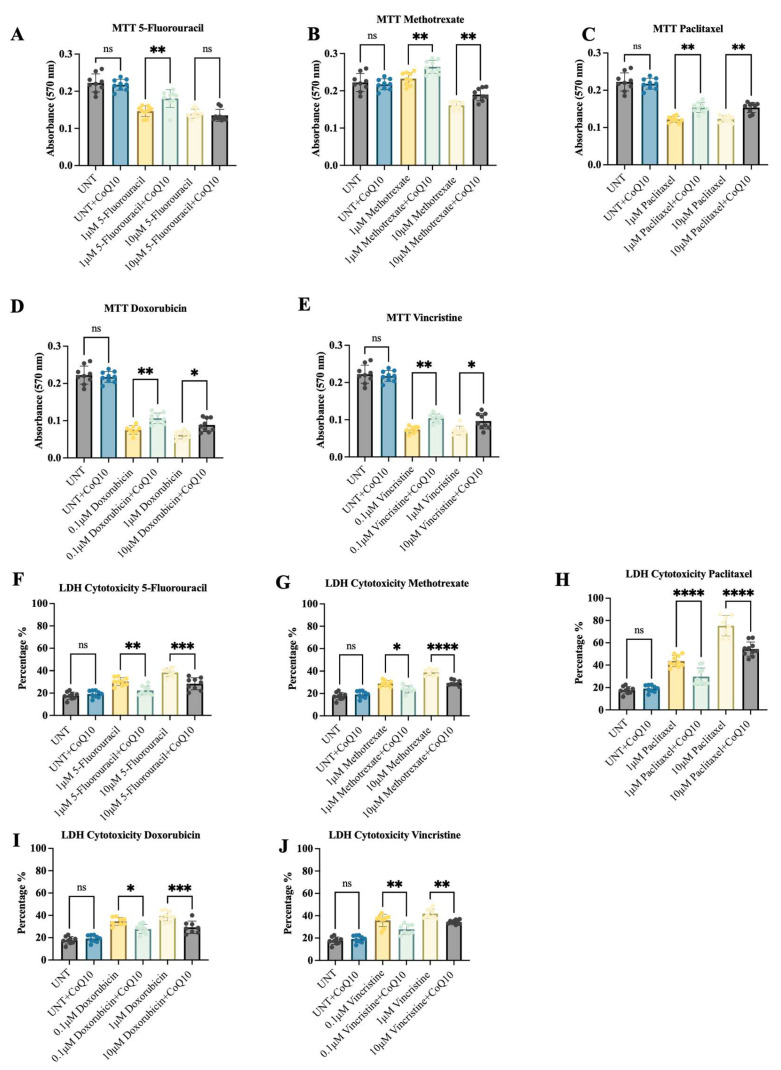
Cell viability analysis in iPSC-MNPs treated with CoQ10 and chemotherapeutic drugs measured using MTT and LDH cytotoxicity assays. Absorbance values were measured 48 h following treatment with CoQ10 (10 μM) in combination with varying concentrations of (**A**,**F**) 5-Fluorouracil, (**B**,**G**) Methotrexate, (**C**,**H**) Paclitaxel, (**D**,**I**) Doxorubicin and (**E**,**J**) Vincristine. Untreated iPSC-MNPs and those treated with 10 μM CoQ10 alone served as controls. Data are expressed as mean ± SE from three independent experiments. ns = not significant, * *p* ≤ 0.05, ** *p* ≤ 0.01, *** *p* ≤ 0.001, **** *p* ≤ 0.0001, and UNT = untreated control.

CoQ10 exposure alone had no significant effect on iPSC-MNPs viability (Figure 2A–E). However, when combined with 5-fluorouracil, an enhancement in viability was observed at the 1 μM (but not at 10 μM) concentration of 5-fluorouracil (Figure 3A). In comparison, CoQ10 treatment showed enhanced viability across all tested concentrations of methotrexate (Figure 3B), paclitaxel (Figure 3C), doxorubicin (Figure 3D) and vincristine (Figure 3E). Overall, the results indicate that CoQ10 exerts protective effects on MNPs exposed to neurotoxic chemotherapeutic agents, as evidenced by MTT analysis.

To confirm these findings, LDH release was examined as a marker of membrane damage and cytotoxicity. Consistent with the viability data, CoQ10 alone had no effect on LDH levels, but, in combination with chemotherapeutic agents, it significantly decreased LDH release in iPSC-MNPs exposed to chemotherapeutic compounds (Figure 3F–J). The decrease was most evident with paclitaxel (Figure 3H), while 5-fluorouracil (Figure 2F), methotrexate (Figure 3G), doxorubicin (Figure 3I), and vincristine (Figure 3J) showed moderate but significant reductions. Together, the results from both MTT and LDH demonstrate that CoQ10 improves iPSC-MNPs cell viability.

### 2.4. CoQ10 Effect on Chemotherapy-Induced ROS Levels in iPSC-MNPs

**Figure 4:**
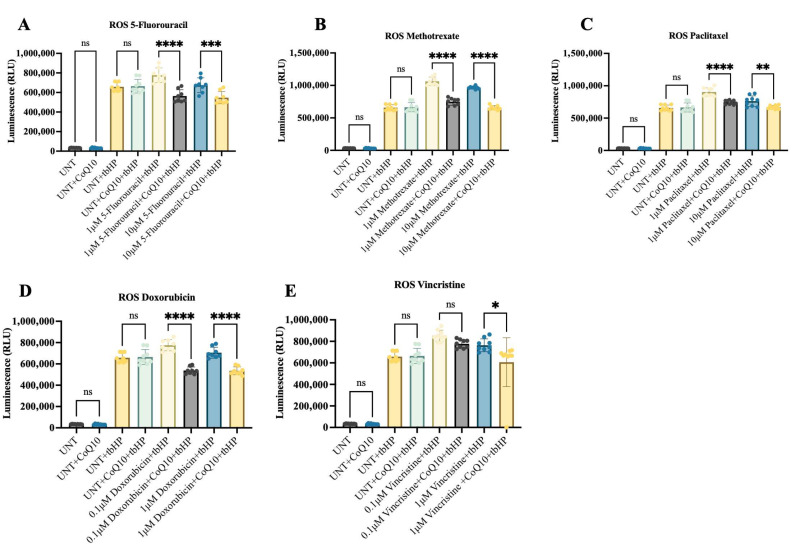
ROS measurement in iPSC-MNPs treated with CoQ10 and chemotherapeutic drugs. Statistical analysis of luminescence-based detection of ROS was performed after treatment with 10 μM CoQ10 in combination with different concentrations of (**A**) 5-Fluorouracil, (**B**) Methotrexate, (**C**) Paclitaxel, (**D**) Doxorubicin and (**E**) Vincristine. iPSC-MNPs treated with 10 μM CoQ10 alone were used as treatment control. For each drug, the following conditions were tested: untreated control (UNT, vehicle), UNT + CoQ10 (10 μM), UNT + tbHP (200 μM, positive control for ROS induction), and UNT + tbHP + CoQ10 (10 μM). Data are presented as mean ± SE from three independent experiments. Statistical significance is indicated by ns = not significant, * *p* ≤ 0.05, ** *p* ≤ 0.01, *** *p* ≤ 0.001, and **** *p* ≤ 0.0001. UNT = untreated control.

ROS production is one of the mechanisms by which chemotherapeutic drugs precipitate neurotoxicity [4,23]. iPSC-MNPs were divided into two groups: one treated with chemotherapeutic agents in combination with CoQ10 supplementation alone, and another group was treated with tbHP, a positive control used as an ROS generator, to further investigate the protective effects of CoQ10 under oxidative stress conditions. While our results demonstrated no significant effects of CoQ10 alone on ROS levels of iPSC-MNPs, a consistent protective effect of CoQ10, marked by a significant reduction in ROS levels, was observed in all tbHP-untreated and -treated cells across all investigated chemotherapy agents (Figure 4A–E). These findings suggest that CoQ10 attenuates chemotherapy-induced ROS generation under oxidative stress conditions.

### 2.5. Effect of CoQ10 on Mitochondrial Membrane Potential (MMP) in Chemotherapy-Treated iPSC-MNPs

**Figure 5:**
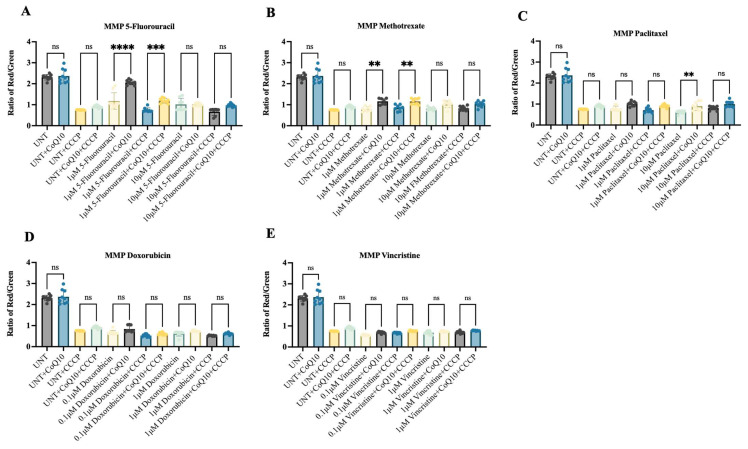
Mitochondrial membrane potential analysis in iPSC-MNPs treated with CoQ10 and chemotherapeutic drugs. MMP was assessed using JC-1 staining, where the red/green fluorescence intensity ratio reflects mitochondrial membrane polarization. Cells were treated with 10 μM CoQ10 in combination with varying concentrations of (**A**) Fluorouracil, (**B**) Methotrexate, (**C**) Paclitaxel, (**D**) Doxorubicin, and (**E**) Vincristine. iPSC-MNPs treated with 10 μM CoQ10 alone served as a control. For each drug, the following conditions were tested: untreated control (UNT, vehicle), UNT + CoQ10 (10 μM), UNT + CCCP (50 μM, positive control for mitochondrial disruption), and UNT + CCCP + CoQ10 (10 μM). Data are presented as mean ± SE from three independent experiments. Statistical significance is indicated by ** *p* ≤ 0.01, *** *p* ≤ 0.001, and **** *p* ≤ 0.0001. UNT = untreated control.

Mitochondrial Potential (MMP), as an indicator of mitochondrial integrity, was assessed using the JC-1 assay. Treatment with CoQ10 alone, in the absence of chemotherapeutic agents, had no significant effect on the red/green fluorescence ratio. In contrast, co-treatment with 10 μM CoQ10 and the chemotherapeutic agents produced variable effects on the red/green ratio of iPSC-derived MNPs (Figure 5). The most pronounced improvement was observed with 5-fluorouracil and methotrexate (Figure 5A and Figure 5B, respectively), particularly at the lower concentration of 1 μM. In contrast, paclitaxel exhibited only a modest rescue effect (Figure 5C). Notably, CoQ10 did not provide measurable protection of the mitochondrial membrane against doxorubicin (Figure 5D) or vincristine (Figure 5E) at any of the concentrations tested. Additionally, we introduced CCCP, a well-known disruptor of mitochondrial membrane potential (MMP), into the experimental system. The addition of CCCP to each chemotherapeutic further reduced MMP; interestingly, subsequent treatment with CoQ10 improved mitochondrial function only in cells exposed to 5-fluorouracil and methotrexate (Figure 5A,B) at the lower concentration of 1 μM. Overall, these results suggest that CoQ10 may alleviate chemotherapy-induced mitochondrial distress in a drug- and dose-dependent manner.

## 3. Discussion

Several common cancer treatments cause nerve damage, adding to the overall burden experienced by patients. This study addressed this challenge by evaluating the neuroprotective potential of CoQ10 using human iPSC-derived neuronal cells. Our results demonstrated that CoQ10 consistently improved neuronal viability, reduced intracellular ROS levels, and preserved mitochondrial membrane potential following exposure to five widely used chemotherapeutics. These findings are consistent with CoQ10’s biological role in supporting mitochondrial function and reducing oxidative stress, two processes known to contribute to the pathogenesis of CIPN. CoQ10 is a vital component of the mitochondrial respiratory chain, where it supports ATP synthesis and acts as a powerful intracellular antioxidant [24]. It is synthesized endogenously and can also be obtained through the diet, playing a key role in protecting mitochondrial membranes and other cellular structures from oxidative damage under stress conditions [11,12,13,14,15].

Several studies have shown that CoQ10 supplementation restores mitochondrial function and reduces oxidative injury in neuronal cells [25,26,27]. While these studies highlighted CoQ10’s protective role in neuronal systems, our work builds on them by assessing its effects in a human iPSC-MNPs model exposed to clinically relevant chemotherapeutic agents. It has also been reported to exert anti-inflammatory effects in models of neurodegeneration, attenuating neuronal inflammation and injury [14,28,29]. Together with our findings, this evidence suggests that CoQ10 exerts potential neuroprotective effects through a combination of mitochondrial support and antioxidant action.

Conventional models used to study CIN, such as rodent systems and immortalized cell-lines, often fail to accurately replicate human neuronal physiology or predict clinical responses. These limitations hinder the discovery of effective neuroprotective strategies for conditions like CIPN. To address this gap, we employed a human iPSC-derived neuronal model, which offers several key advantages, including species-specific relevance, reproducible differentiation protocols, and accessibility for high-content functional assays. In our study, three iPSC lines were confirmed to be genetically stable and pluripotent, and all successfully differentiated into motor neurons expressing stage-specific neuronal markers. This approach is consistent with previous research demonstrating the utility of iPSC-based systems in modeling neurological disorders and drug responses [22,30,31]. By providing a scalable platform that closely reflects human neuronal biology, our model supports its use in future neurotoxicity and neuroprotection research.

Using these human iPSC-derived neurons, we demonstrated that CoQ10 provided consistent protection against CIN across multiple functional endpoints. When administered alongside chemotherapeutic agents, CoQ10 significantly improved motor neuron viability, preserved mitochondrial membrane potential in a drug- and dose-dependent manner, and reduced intracellular ROS levels. Importantly, the chemotherapeutic agents tested in this study, 5-fluorouracil (antimetabolite), methotrexate (antimetabolite), paclitaxel (microtubule poison), doxorubicin (topoisomerase II inhibitor and ROS generator), and vincristine (microtubule poison), exert their cytotoxicity via distinct mechanisms, suggesting that the neuroprotective effects of CoQ10 are not limited to a single class of drugs but may reflect a broader mitochondrial and oxidative stress-targeted protective action. Notably, CoQ10 alone had no significant impact on baseline neuronal viability or mitochondrial function in our model, suggesting that its actions are specifically triggered under chemostress conditions. It should be noted, however, that loss of MMP can result both from the direct impact of chemotherapeutic agents on mitochondria and indirectly because of cell death. Our use of JC-1 staining detects early mitochondrial depolarization, supporting a direct effect on mitochondrial function, although later stages of cell death may also contribute to the observed decline. These findings are in line with prior reports showing that CoQ10 enhances mitochondrial respiration, stabilizes membrane potential, and limits oxidative damage [14,32,33]. Supporting evidence for the neuroprotective potential of CoQ10 has also been reported in vivo, where it reversed chemotherapy-induced cognitive impairment in mice, reduced oxidative stress, increased antioxidant enzyme levels, and modulated inflammatory markers [34,35,36,37,38], highlighting its broader relevance for neuroprotection in cancer treatment.

This study focused on a single CoQ10 concentration administered concurrently with chemotherapeutic agents in vitro. Future work may benefit from exploring dose–response relationships and alternative administration schedules, including pre- and post-treatment strategies, to better reflect clinical scenarios and optimize therapeutic outcomes. While our human iPSC-derived model provides valuable insight, translating these findings into more complex physiological systems will require complementary in vivo validation, which provided a clear assessment of its protective potential under co-exposure conditions. We also acknowledge that our findings suggest CoQ10 may not strongly influence baseline chemotherapy-induced ROS levels, but its protective effect became evident under conditions of additional oxidative stress inflicted by tbHP treatment. An additional limitation is that the ROS detection kit used in this study measures general ROS and does not specifically reflect the extent of lipid peroxidation. Since CoQ10 may influence lipid peroxidation, future studies employing lipid peroxidation-specific assays will be needed to confirm this effect. 

Taken together, our findings suggest that CoQ10 may offer a practical and biologically grounded approach to protect neurons from chemotherapy-induced damage. As CIN continues to limit treatment outcomes and diminishes quality of life, the need for accessible and well-tolerated neuroprotective strategies remains critical. By combining a human-relevant neuronal model with clinically used chemotherapeutic agents, this study contributes to translational efforts aimed at reducing the neurological burden of cancer therapy and improving patient care.

## 4. Materials and Methods

All chemicals and reagents used in this study were sourced from Sigma-Aldrich/Merck (Gillingham, UK), etc., unless specified otherwise. Three iPSC lines were previously generated from human dermal fibroblast (HDF) biopsies obtained from three healthy Jordanian donors; one male and two females and were named JUCTCi011-A (iPSC-01), JUCTCi010-A (iPSC-02) and JUCTCi010-B (iPSC-03), respectively [19,21,39]. Briefly, HDFs at Passage 3 were reprogrammed using the CytoTune-iPS 2.0 Sendai Reprogramming Kit (Thermo Fisher Scientific, Waltham, MA, USA), following the manufacturer’s instructions. The resulting iPSCs were expanded under feeder-free conditions on Matrigel-coated plates and maintained in mTeSR™1 medium (STEMCELL Technologies, Vancouver, BC, Canada). To confirm the sterility of the iPSC lines, we assessed the absence of mycoplasma using the MycoAlert Detection Kit (Lonza, Basel, Switzerland) following the manufacturer’s instructions.

### 4.1. Immunophenotyping of iPSCs

To assess pluripotency, iPSC lines were harvested with 0.5 mM EDTA and analyzed by flow cytometry for the pluripotency markers NANOG and TRA-1-60. Briefly, cells were fixed for 10 min at room temperature (RT) in 2% paraformaldehyde (Merck, Sigma-Aldrich, St. Louis, MO, USA), then washed with 1X phosphate-buffered saline (1X PBS). Permeabilization was performed using 100% ice-cold methanol. The resulting pellet was then resuspended in 1 mL ice-cold methanol and incubated at −20 °C for 2 h. Following two PBS washes, cells were resuspended in 400 μL of 2% bovine serum albumin (BSA) in PBS staining buffer (BD Biosciences, San Jose, CA, USA). Freshly prepared antibodies were added to 100 μL aliquots of each cell suspension and incubated in the dark with continuous shaking for 40 min (Supplementary Table S1). Samples were acquired on a BD FACS Canto II and analyzed using BD FACSDiva software version 8 (BD Biosciences, San Jose, CA, USA) [19,39].

### 4.2. hiPSCs Karyotyping

To evaluate chromosomal stability, iPSCs were treated with colcemid (10 μg/mL; Euroclone, Pero, Italy) and incubated for 16–18 h, to arrest cells in metaphase. Cells were then exposed to a hypotonic solution (0.075 M KCl; Euroclone, Pero, Italy) to induce chromosome swelling followed by washing and fixation using Carnoy’s solution (3:1 Methanol: Acetic Acid). Fixed cells were dropped onto a clean microscopic slide from a height of a few centimeters and allowed to spread. The slides were then analyzed using an Imager D2 microscope (Carl Zeiss Microscopy GmbH, Jena, Germany) and chromosomal images were captured with the Vision Karyo 3.1 imaging system (West Medica, Vienna, Austria).

### 4.3. Neuronal Differentiation

Neuronal differentiation was initiated by replacing confluent iPSC cultures with a basal medium (1:1 DMEM/F12 and Neurobasal) supplemented with N2, B27, ascorbic acid, GlutaMAX, and antibiotic-antimycotic solution (all from Gibco, Thermo Fisher Scientific, Waltham, MA, USA). For induction, 3 μM CHIR-99021, 2 μM DMH1, and 2 μM SB431542 were added (STEMCELL Technologies, Vancouver, BC, Canada). Cells were maintained under standard conditions (21% O_2_, 37 °C, 5% CO_2_) with medium changes every other day. On day 7, cells were differentiated into neural progenitors (Day 7 NPCs), then dissociated with Accutase (Gibco, Thermo Fisher Scientific, Waltham, MA, USA) and replated at a 1:3 ratio in medium containing retinoic acid (RA), puromorphamine (Pur), CHIR-99021, DMH1, and SB431542 to generate motor neuron progenitors (Day 12 MNPs). For motor neuron differentiation, Day 12 NPCs were detached with Accumax and maintained in the same medium with 0.5 μM RA and 0.1 μM puromorphamine for 6 days, with medium changes every two days. On day 18, MNPs were detached using Accumax, dissociated into single cells, and plated onto pre-coated Matrigel (Corning, Corning, NY, USA) six-well plates. For the final 30 days, MNs were cultured in basal medium containing 0.5 μM RA, 0.1 μM puromorphamine, and 0.1 μM Compound E (STEMCELL Technologies, Vancouver, BC, Canada) for 10 days. Additionally, brain-derived neurotrophic factor (BDNF) and glial cell-derived neurotrophic factor (GDNF) (both from R&D Systems, Minneapolis, MN, USA, 10 ng/mL each) were added to promote survival and neurite outgrowth [21,40].

### 4.4. Immunofluorescence Staining of Neurons

Immunostaining was used to confirm neuronal differentiation by detecting markers like TUJ1, NESTIN, PAX6, and SOX1. Briefly, 60,000 cells were plated on coverslips, fixed with 3% formaldehyde, and blocked/permeabilized using 10% NGS and 0.3% Triton X-100. Cells were incubated overnight at 4 °C with primary antibodies (1:500 dilution), followed by secondary Alexa Fluor™ 488 or 546-conjugated antibodies (1:500). Day 18 MNPs were stained with PAX6:NESTIN, SOX1:TUJ1, and ISL1:SMI32, while Day 30 MNs were stained with PAX6:TUJ1 and ISL1:TUJ1. DAPI was used for nuclear staining, and imaging was performed using a Zeiss time-lapse microscope. A comprehensive list of antibodies is provided in Supplementary Table S1.

### 4.5. Chemotherapy Compounds Preparation

To evaluate the neuroprotective effect of CoQ10, five chemotherapeutic compounds were used in this study: 5-fluorouracil (384 mM), methotrexate (55 mM), paclitaxel (7 mM), doxorubicin (3.44 mM) and vincristine (1.2 mM). The chemotherapeutic compounds were resuspended in 1:1000 DMSO (vehicle). Final concentrations in the culture media were as follows: 5-fluorouracil, doxorubicin and methotrexate at 0 μM, 1 μM and 10 μM; vincristine and paclitaxel at 0 μM, 0.1 μM and 1 μM [41] CoQ10 was prepared as a 10 mM stock solution in DMSO and used at a final concentration of 10 μM [42,43]. These concentrations were used simultaneously to assess the potential protective effects of CoQ10 when combined with the chemotherapeutic compounds functional assays including, MTT, ROS, and JC-1assays for cell viability, oxidative stress, and mitochondrial potential, respectively. Only day-18 MNPs were used in subsequent experiments.

### 4.6. Cell Viability Assay (MTT)

To access cell viability, day 18 MNPs, were seeded at a density of 30,000 cells per well in 96-well plates and treated with 10 μM CoQ10 treatment in the presence or absence of chemotherapeutic agents. Untreated cells (UNT; vehicle control, 1:1000 DMSO) were used as the negative control. Cell viability was assessed by adding 10 μL per well of MTT reagent [3-(4,5-dimethylthiazol-2-yl)-2,5-diphenyltetrazolium bromide; ATCC, Manassas, VA, USA]. Following a 3 h incubation under standard culture conditions (37 °C, 5% CO_2_), 110 μL of solubilization/stop solution was added to each well, and the plates were incubated for an additional 30 min at 37 °C and 5% CO_2_. Absorbance was measured at 570 nm using a Cytation 5 multimode microplate reader (BioTek Instruments, Winooski, VT, USA), and data were analyzed with Gen5 software v3.x (BioTek Instruments, Winooski, VT, USA).

### 4.7. LDH Assay

Lactate dehydrogenase (LDH) release was measured as an indicator of membrane integrity and compound-induced cytotoxicity. Day 18 MNPs were seeded at a density of 50,000 cells per well in 96-well plates and exposed to 10 μM CoQ10 in combination with/without chemotherapeutic agents, and culture supernatants were collected at the end of treatment. Untreated cells (UNT; vehicle control, 1:1000 DMSO) were used as the negative control. LDH activity in the culture supernatant was measured using the CytoTox 96^®^ Non-Radioactive Cytotoxicity Assay (Promega, Madison, WI, USA), which quantifies the enzymatic conversion of lactate to pyruvate coupled to the formation of a red formazan product. Absorbance was recorded at 490 nm using a Cytation 5 multimode plate reader (BioTek Instruments, Winooski, VT, USA). Values were normalized to untreated controls to calculate the percentage of LDH release.

### 4.8. Reactive Oxygen Species (ROS) Assay

Intracellular ROS levels in day 18 MNPs treated with 10 μM CoQ10 in combination with varying concentrations of chemotherapeutic compounds were measured using the Total Reactive Oxygen Species (ROS) Assay Kit (Abcam, Cambridge, UK; ab113851), following the manufacturer’s instructions. Briefly, MNPs were seeded at a density of 30,000 cells/well in 96-well black-walled plates (Costar^®^, Corning Inc., Corning, NY, USA) using day 18 neuronal media as described above. Cells were treated with chemotherapeutic compounds as described and incubated for 48 h at 37 °C in a humidified atmosphere with 5% CO_2_. Untreated cells (UNT; vehicle control, 1:1000 DMSO) were used as the negative control. Following treatment, the medium was removed, and 50 μL of 1X ROS stain solution was added to each well. The stain solution was prepared by diluting 10 μL of the 500X stock into 5 mL of 1X PBS in 10X binding buffer. Plates were incubated for 60 min under standard conditions (37 °C, 5% CO_2_). In control wells, 200 μM tert-butyl hydrogen peroxide (tbHP, Sigma-Aldrich, St. Louis, MO, USA) in the same buffer used in ROS was added after 30 min, following the manufacturer’s guidelines. Fluorescence was then measured using a Cytation 5 multimode microplate reader (BioTek Instruments, Winooski, VT, USA) at 488 nm excitation and 520 nm emission. Data analysis was performed using Gen5 software (BioTek Instruments, Winooski, VT, USA).

### 4.9. Mitochondrial Membrane Potential (MMP)

To assess mitochondrial membrane potential (Δψm), day 18 treated MNPs were stained using the MitoProbe™ JC-1 Assay Kit (Invitrogen, Thermo Fisher Scientific, Waltham, MA, USA). Briefly, cells were seeded at a density of 30,000 cells/well in 96-well black-walled plates (Costar®, Corning Inc., Corning, NY, USA) in day 18 neuronal media and treated as described above. Treated cells were then incubated with 45 μL of 20 μM JC-1, prepared in the same binding buffer used for ROS assay, for 60 min at 37 °C in a humidified incubator with 5% CO_2_. For positive control wells, 45 μL of carbonyl cyanide 3-chlorophenylhydrazone (CCCP, 50 μM; Sigma) was added 30 min after JC-1 incubation, and cells were incubated for an additional 30 min. After staining, fluorescence was measured using the Cytation 5 multimode plate reader. JC-1 monomers (green; depolarized mitochondria) were detected at 485 nm excitation/528 nm emission, while JC-1 aggregates (red; polarized mitochondria) were detected at 535 nm excitation/590 nm emission. Mitochondrial membrane potential (Δψm) was quantified as the ratio of red (aggregates) to green (monomers) fluorescence using Gen5 software (BioTek Instruments, Winooski, VT, USA).

### 4.10. Statistical Analysis

All data were analyzed using GraphPad Prism v9.1.0 (GraphPad Software, La Jolla, CA, USA). Statistical significance was determined using one-way ANOVA, and two-way ANOVA, followed by Bonferroni post hoc analysis where appropriate. Results are presented as mean ± standard error (SE). All experiments were performed independently in triplicate, with each experiment including technical triplicates. Statistical significance was considered at *p* ≤ 0.05, with significance levels denoted as follows: *p* ≤ 0.05 (*), *p* ≤ 0.01 (**), *p* ≤ 0.001 (***), and *p* ≤ 0.0001 (****).

## 5. Conclusions

This study is the first to investigate the neuroprotective effects of CoQ10 against CIN using human iPSC-derived neurons. By integrating five widely used chemotherapeutic agents, the work offers a clinically relevant and human-specific platform for understanding neuronal vulnerability during cancer treatment. The model proved effective in capturing key neurotoxic effects and evaluating protective interventions. CoQ10 consistently improved neuronal viability, reduced oxidative stress, and preserved mitochondrial function under chemotherapeutic stress. These findings highlight its potential as a targeted neuroprotective agent and lay the groundwork for future preclinical studies aimed at reducing the neurological burden of cancer therapy.

## Data Availability

Data is contained within the article and Supplementary Materials.

## Supplementary Materials

The supporting information can be downloaded at: https://www.mdpi.com/article/10.3390/ijms26199647/s1.
