# Supplementary material for: Coenzyme Q10 Ameliorates Chemotherapy-Induced Neurotoxicity in iPSC-Derived Neurons by Reducing Oxidative Stress
Source: Int J Mol Sci. 2025 Oct 2;26(19):9647. doi: 10.3390/ijms26199647 (PMC12524460; doi:10.3390/ijms26199647)
Supplement: Supplementary file 1 [file ijms-26-09647-s001.zip › ijms-3763590-supplementary.pdf]

International Journal of
Molecular Sciences

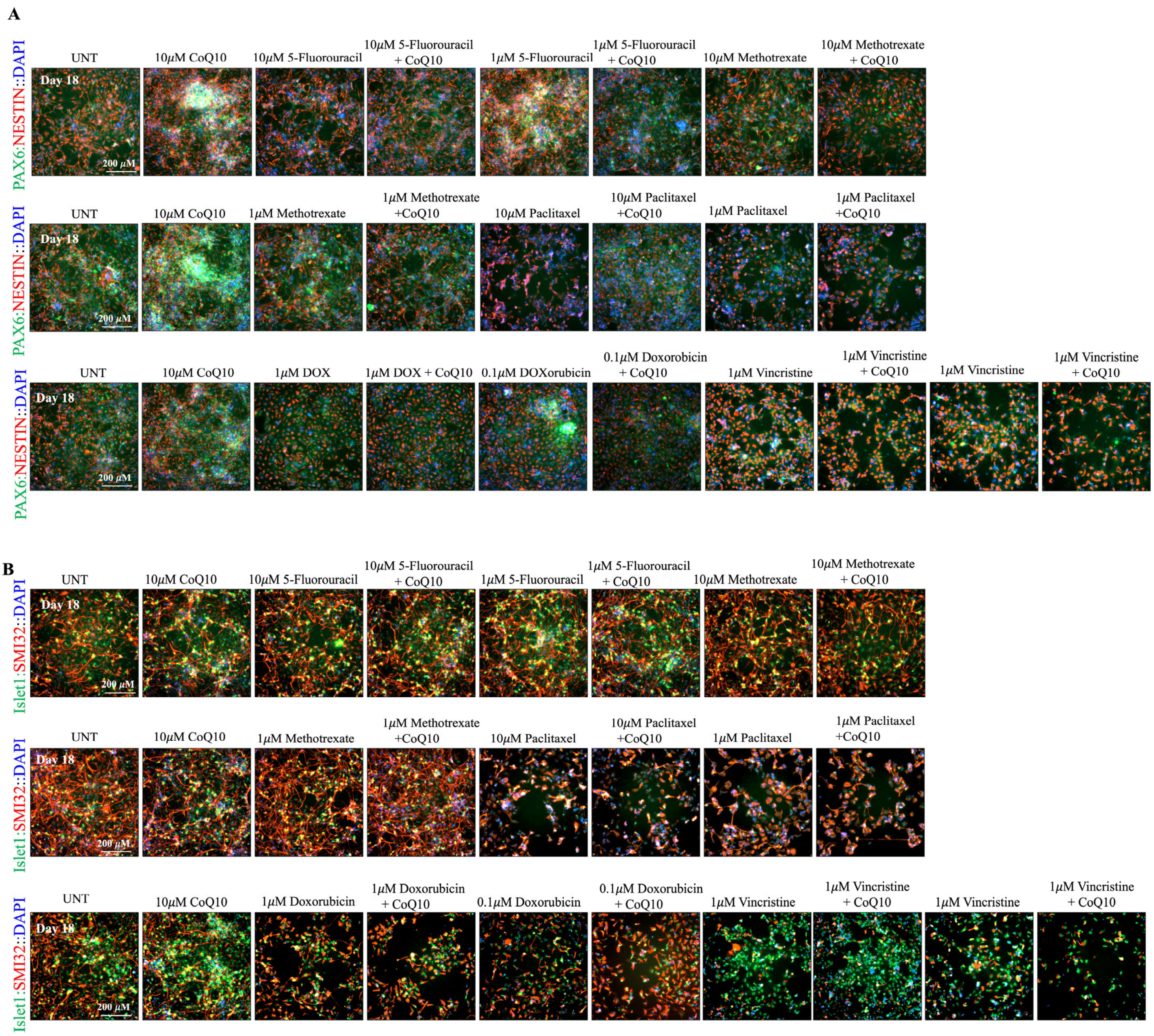

Supplementary Figure S1. Immunocytochemical characterization of iPSC-derived neuronal cells.
(A) Staining of iPSC-derived neural progenitors at day 18 following CoQ10 and chemotherapy treatments, showing PAX6 (green) and Nestin (red) expression, confirming neural lineage specification. Nuclei were counterstained with DAPI (blue). (B) Staining of iPSC-derived neurons at day 18 after CoQ10 and chemotherapy treatments, showing ISL1 (green) and SMI32 (red) expression, indicating acquisition of a motor neuron phenotype. Nuclei were counterstained with DAPI (blue). Scale bars: 200 μm.

Table S1

Antibody (Ab) / Isotype	Dilution	Cat.No
Alexa Fluor® 647 anti-Nanog Antibody	1:500	674010
AF-647- Alexa Fluor® 647 Mouse IgG1, κ Isotype Ctrl (ICFC) Antibody	1:50	400136
Alexa Fluor® 488 anti-human TRA-1-60-R Antibody	1:50	330614
Alexa Fluor® 488 Mouse IgM, κ Isotype Ctrl Antibody	1:1000	401617
Anti-SOX1 antibody	1:500	ab242125
beta-3 Tubulin (TUJ1) Monoclonal Antibody	1:1000	ab7751
Islet1 Recombinant Rabbit Monoclonal Antibody (	1:500	ab109517
Nestin Monoclonal Antibody	1:500	ab18102
PAX6 Recombinant Rabbit Monoclonal Antibody (SD08-31)	1:500	ab195045
Goat anti-Rabbit IgG (H+L) Highly Cross-Adsorbed Secondary Antibody, Alexa Fluor™ 546	1:500	A-11035
Goat anti-Mouse IgG1 Cross-Adsorbed Secondary Antibody, Alexa Fluor™ 488	1:500	A-21121
